# Cytokinin-Dependent Control of *GH3* Group II Family Genes in the *Arabidopsis* Root

**DOI:** 10.3390/plants8040094

**Published:** 2019-04-08

**Authors:** Emanuela Pierdonati, Simon Josef Unterholzner, Elena Salvi, Noemi Svolacchia, Gaia Bertolotti, Raffaele Dello Ioio, Sabrina Sabatini, Riccardo Di Mambro

**Affiliations:** 1 Dipartimento di Biologia e Biotecnologie, Laboratory of Functional Genomics and Proteomics of Model Systems, Università di Roma, Sapienza-via dei Sardi, 70–00185 Rome, Italy; emanuela.pier@gmail.com (E.P.); unterholzner.simonjosef@gmail.com (S.J.U.); elena.salvi2@gmail.com (E.S.); svolacchia.noemi@gmail.com (N.S.); gaia.bertolotti@uniroma1.it (G.B.); raffaele.delloioio@uniroma1.it (R.D.I.); sabrina.sabatini@uniroma1.it (S.S.); 2 Department of Biology, University of Pisa-via L. Ghini, 13–56126 Pisa, Italy

**Keywords:** *GRETCHEN HAGEN 3 (GH3) IAA-amido synthase group II*, root apical meristem, auxin, cytokinin, lateral root cap, auxin minimum, auxin conjugation

## Abstract

The *Arabidopsis* root is a dynamic system where the interaction between different plant hormones controls root meristem activity and, thus, organ growth. In the root, a characteristic graded distribution of the hormone auxin provides positional information, coordinating the proliferating and differentiating cell status. The hormone cytokinin shapes this gradient by positioning an auxin minimum in the last meristematic cells. This auxin minimum triggers a cell developmental switch necessary to start the differentiation program, thus, regulating the root meristem size. To position the auxin minimum, cytokinin promotes the expression of the *IAA-amido synthase group II* gene *GH3.17*, which conjugates auxin with amino acids, in the most external layer of the root, the lateral root cap tissue. Since additional *GH3* genes are expressed in the root, we questioned whether cytokinin to position the auxin minimum also operates via different *GH3* genes. Here, we show that cytokinin regulates meristem size by activating the expression of *GH3.5* and *GH3.6* genes, in addition to *GH3.17*. Thus, cytokinin activity provides a robust control of auxin activity in the entire organ necessary to regulate root growth.

## 1. Introduction

Organ growth in plants is supported by the meristems, regions providing a reservoir of undifferentiated cells whose activity depends on the stem cell niche [1]. In the root, the stem cells daughters proliferate establishing the division zone of the meristem and, more distally from the root tip along the longitudinal axis, those cells differentiate generating the differentiation zone [2,3,4,5]. The boundary between proliferating and differentiating cells is called transition zone (TZ). The position of this cell boundary depends on the coordinated activity of the stem cell niche, the division zone, and the differentiation zone. The activities of these zones are controlled by a dynamic equilibrium between cell division and cell differentiation. The regulation of this equilibrium results in a shoot-ward or a root-ward shift of the TZ position along the root longitudinal axis [2,3,4,6]. The position of the TZ depends on the antagonistic interaction between cytokinin and auxin hormones [7,8]. It has been demonstrated that cytokinin controls TZ localization by positioning an auxin minimum specifically in the last meristematic cells of each root tissue [9]. In particular, cytokinin through the primary cytokinin response transcription factor *ARABIDOPSIS* RESPONSE REGULATOR 1 (ARR1), positively regulates the expression of the *Aux/IAA SHORT HYPOCOTYL 2* (*SHY2*) gene, which in turn negatively controls the polar auxin efflux carriers *PIN1, PIN3* and *PIN7* genes at the vascular tissue TZ. At the same time, ARR1 positively regulates the expression of the *IAA-amino synthase* of the *GH3* Group II gene family *GRETCHEN HAGEN 3.17* (*GH3.17*) [8,9].

The roots of *Arabidopsis thaliana* can be represented as a series of concentric cylinders where the vascular bundles lie in the center [1,2,3,4]. In the radial axis of the root, the lateral root cap (LRC) represents the most external tissue that surrounds all tissues of the root meristem [1,2,3,4]. The LRC serves to facilitate root penetration in the soil, it acts as a physical protective barrier of the root meristem, and it plays an important role in meristem maintenance [10,11,12,13,14,15,16,17]. It was previously demonstrated that a molecular mechanism acting specifically in the LRC controls root meristem size and, thus, root growth, by positioning the TZ [17]. In particular, ARR1, besides *GH3.17*, regulates auxin levels by promoting the transcription of the auxin intracellular transporter *PIN-FORMED 5* (*PIN5*) gene [17]. GH3.17 irreversibly conjugates free auxin with amino acids specifically in the LRC cells, thus, promoting hormone inactivation, whereas PIN5 operates on auxin intracellular homeostasis mediating auxin compartmentalization in the endoplasmic reticulum. As a result, the LRC acts as an auxin sink where the regulation of auxin levels, controlled by the cytokinin activity, influences auxin distribution within the entire meristem regulating root meristem size and, thus, root growth [17].

Due to the importance of the tissue-specific activity of cytokinin in the LRC, we question whether cytokinin controls meristem size from this tissue by acting on additional genes. It has been already reported that the induction of cytokinin activity in the LRC regulates the expression of *GH3.5*, *GH3.6* and *GH3.9* genes [17], members of the *GH3* Group II gene family [18,19,20].

Here we show that *GH3.5* and *GH3.6* genes are expressed in the LRC and that their expression is cytokinin-dependent. We also show that those genes, similarly to *GH3.17*, are involved in meristem size regulation. These findings highlight the pivotal role of cytokinin in localizing a strong auxin inactivation process in the LRC to regulate meristem activity.

## 2. Results

In order to unveil cytokinin-dependent mechanisms acting in the LRC to control meristem size, we took advantage of the already published microarray data reporting genes differentially regulated in the LRC in response to ARR1 induction. These data, resulting from the transcriptional profiling of LRC cells upon induction of a constitutive active form of ARR1 (ARR1ΔDDK) in the LRC, revealed that genes belonging to “auxin homeostasis regulation” gene ontology category are highly represented [17]. Interestingly, among these genes, *GH3.5*, *GH3.6*, and *GH3.9* were positively regulated by ARR1 in the LRC [17]. It was demonstrated that GH3.5, GH3.6, and GH3.9 IAA-amido synthases participate in maintaining auxin homeostasis by conjugating amino acids to the hormone [18,19,21,22,23,24] and thereby affect the levels of free auxin molecules that are biologically active and suited for binding to their receptors.

**Figure 1:**
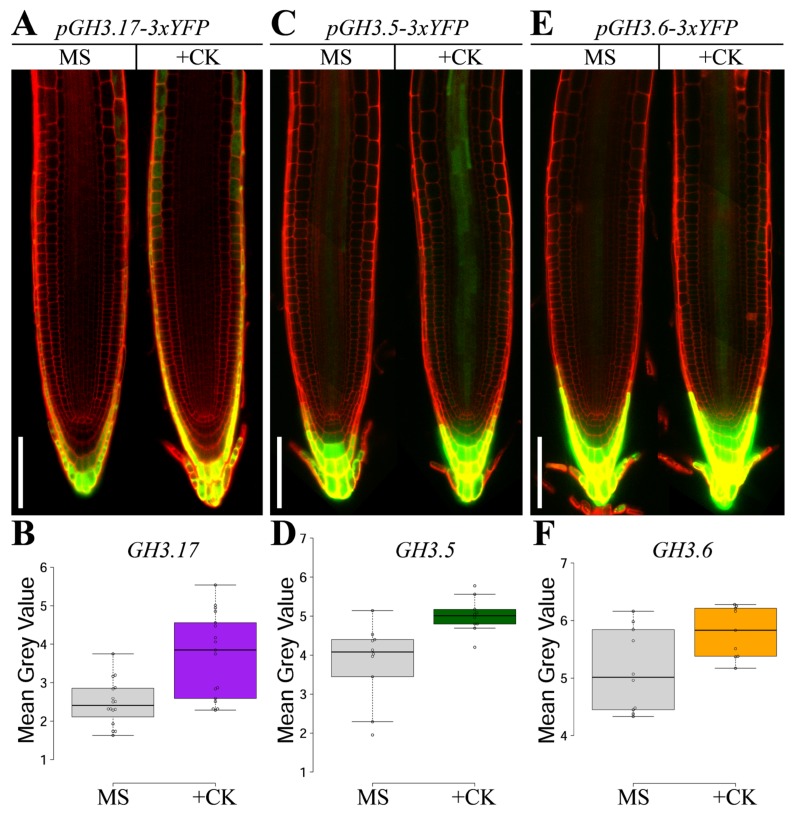
Cytokinin induces *GH3.5* and *GH3.6* expressions. (**A**,**C**,**E**) Confocal images of five days after germination (dag) roots expressing *pGH3.17–3xYFP*, *pGH3.5–3xYFP* and *pGH3.6–3xYFP* constructs untreated (MS) and treated for four hours with 5 μM of cytokinin (+CK) (see Materials and Methods). Scale bar, 100 µm. (**B**,**D**,**F**) *Mean Grey Value* quantification of *pGH3.17–3xYFP*, *pGH3.5–3xYFP* and *pGH3.6–3xYFP* lines untreated (grey) and treated with cytokinin 5μM for four hours (+CK) (purple-*pGH3.17–3xYFP*; green-*pGH3.5–3xYFP*; orange-*pGH3.6–3xYFP* lines, respectively) at 5 dag where center lines show the medians. Box limits indicate the 25th and 75th percentiles as determined by *R* software. Whiskers extend 1.5 times the interquartile range from the 25th and 75th percentiles, data points are plotted as open circles. Statistical significance: (**B**) Two biological replicates. *p*-value < 0.005, Student’s t-test, *n* = 16, 17 sample points, (**D**) two biological replicates. *p*-value < 0.05, Student’s t-test, *n* = 10, 9 sample points, (**F**) *p*-value < 0.005, Student’s t-test, *n* = 10, 10 sample points.

Considering the LRC specific ARR1-dependent positive regulation of *GH3.5*, *GH3.6*, and *GH3.9*, and the LRC specific domain of activity of GH3.17, we thus questioned whether the expression domain of *GH3.5*, *GH3.6*, and *GH3.9* localizes in the LRC. To this end, we generated GREEN FLUORESCENT PROTEIN (GFP) translational fusions of these three *GH3s* (*pGH3.5::GH3.5-GFP*, *pGH3.6::GH3.6-GFP*, and *pGH3.9::GH3.9-GFP* lines, respectively). The GFP signal was undetectable for all those lines (data not shown), most likely because of the low expression of those genes in the root as also previously reported [25]. Therefore, we developed transcriptional fluorescent reporters for each of the *GH3s* using a three-time YELLOW FLUORESCENT PROTEIN (*3xYFP*) fusion (*pGH3.9–3xYFP, pGH3.5–3xYFP*, and *pGH3.6–3xYFP* lines, respectively). Additionally, we also generated a *GH3.17* transcriptional fusion line with the same reporter (*3xYFP*) (*pGH3.17–3xYFP* line) to verify the overlap of the expression domains of the translational and the transcriptional fusions of *GH3.17*. The *pGH3.17–3xYFP* line revealed a localized YFP expression in the more external layer of the LRC and in the differentiated epidermal cells (Figure 1A), resembling that of the *pGH3.17:GH3.17-GFP* translational fusion [9], and upon cytokinin treatment *pGH3.17–3xYFP* expression was significantly increased (Figure 1A,B). Although YFP expression was not detectable in *pGH3.9–3xYFP* line (data not shown), the analysis of *pGH3.5–3xYFP* and *pGH3.6–3xYFP* lines revealed, similarly to *pGH3.17–3xYFP* line, a fluorescent signal in the LRC tissue (Figure 1C,E). Moreover, based on the microarray data [17] and given that cytokinin promotes *GH3.17* expression (Figure 1) [9], we verified if the expression of *GH3.5* and *GH3.6* are responsive to cytokinin analyzing *pGH3.5–3xYFP* and *pGH3.6–3xYFP* lines upon cytokinin treatment. The fluorescence signal of *pGH3.5–3xYFP* and *pGH3.6–3xYFP* was detected in the youngest cells of the outermost LRC layer, in the columella and in the vascular tissues (Figure 1C,E). After four hours of cytokinin treatment, the fluorescence signal of *pGH3.5–3xYFP* and *pGH3.6–3xYFP* lines was significantly increased compared to untreated lines (Figure 1C–F). Furthermore, while *GH3.17* expression was localized in the more external tissues of the root (the LRC and the differentiated epidermal cells), *GH3.5* and *GH3.6* expression was induced also in the vascular tissue (Figure 1C,E). This hinted at the possibility that *GH3.5* and *GH3.6* are involved in the regulation of auxin levels not only in the LRC, where the regulation of auxin levels affects meristem size but also in the vascular tissue, possibly in coordination with the robust auxin flux active in this tissue. 

Taken together, these results corroborate the idea that a cytokinin-dependent mechanism regulates the auxin inactivation process by controlling the expression of several members of the *GH3* Group II gene family. 

**Figure 2:**
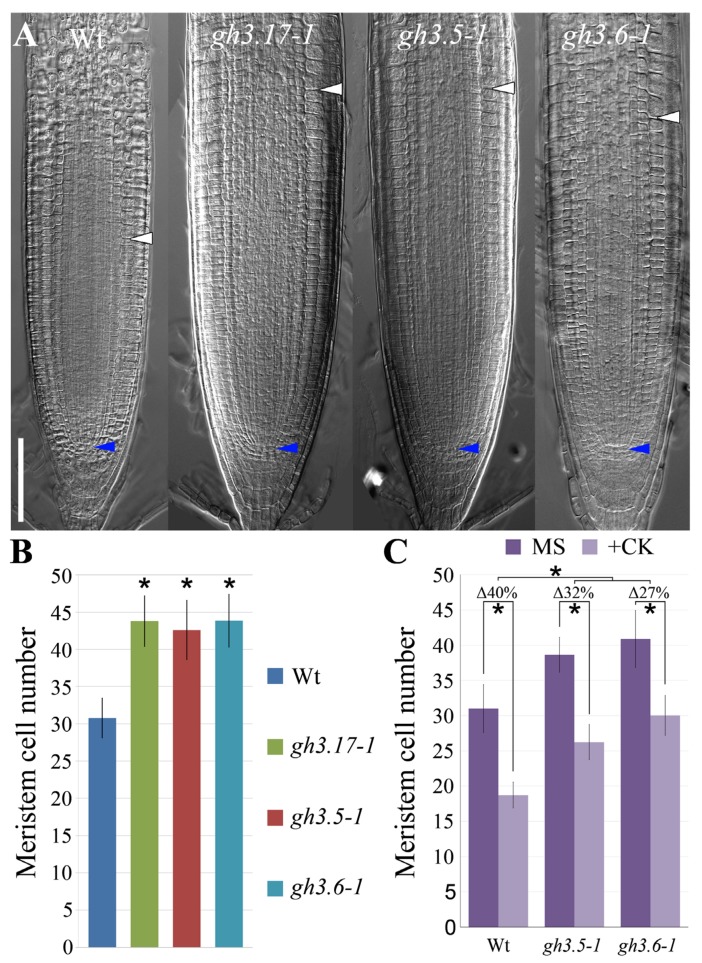
*GH3.5* and *GH3.6* are involved in the control of root meristem size. (**A**) Root meristems at 5 dag of Wt, *gh3.17–1*, *gh3.5–1* and *gh3.6–1* plants. Blue and white arrowheads indicate the quiescent center (QC) and the cortex transition zone (i.e., meristem size), respectively. Scale bar, 100 µm. (**B**) Analysis of meristematic cortical cell number of Wt, *gh3.17–1*, *gh3.5–1*, and *gh3.6–1* plants. Error bars indicate standard deviation (SD). Two biological replicates were performed. Asterisk (*) indicates a significance with a *p*-value < 0.005, Student’s t-test, *n* = 18, 15, 16, 17. (**C**) Analysis of meristematic cortical cell number of Wt, *gh3.5–1* and *gh3.6–1* untreated (MS) plants and after 22 h of cytokinin treatment (+CK) (see *Materials and Methods*). “Δ” indicates the relative decrease percentage in the number of meristematic cells of the cortex after cytokinin treatment. Error bars indicate SD. Two biological replicates were performed. * indicates a significance with a *p*-value < 0.005, Student’s *t*-test, *n*_(MS)_ = 14, 14, 18 and *n*_(+CK)_ = 15, 16, 22.

**Figure 3:**
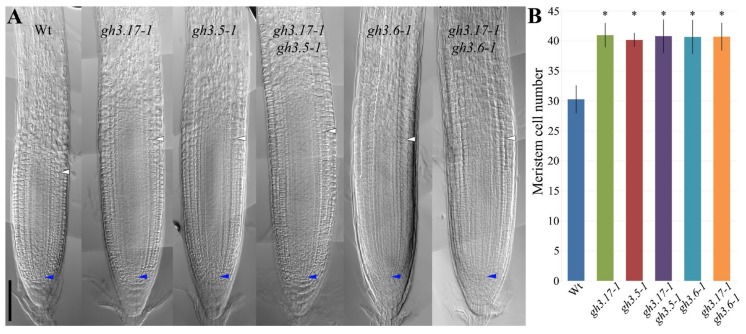
GH3.5, GH3.6, and GH3.17 synergistically act in the control of meristem size. (**A**) Bright field microscopy images of root apical meristems at 5 dag of Wt, *gh3.17–1*, *gh3.5–1*, *gh3.17–1*;*gh3.5–1*, *gh3.6–1* and *gh3.17–1;gh3.6–1* plants. Blue and white arrowheads indicate the QC and the cortex transition zone (i.e., meristem size), respectively. Scale bar, 100 µm. (**B**) Analysis of the number of meristematic cells of the cortex of Wt, *gh3.17–1*, *gh3.5–1*, *gh3.17–1*;*gh3.5–1*, *gh3.6–1* and *gh3.17–1;gh3.6–1* plants. Error bars indicate SD. Two biological replicates were performed. * indicates a significance with a *p*-value < 0.001, Student’s t-test, *n* = 26, 22, 20, 32, 30, 30.

It has been shown that GH3.17 activity in the LRC is necessary and sufficient for the regulation of the meristem size [9,17]. To understand if GH3.5 and GH3.6 are involved in the control of the meristem activity, we analyzed the meristem size of *gh3.5–1* and *gh3.6–1* loss of function mutants. The meristem size is measured taking into account the number of meristematic cells of the cortex tissue [3]. In a similar way to *gh3.17–1* mutant, *gh3.5–1* and *gh3.6–1* mutants showed increased meristem size when compared to wild type plants (Figure 2A,B). These data indicate that GH3.5 and GH3.6 activities together with GH3.17 are required for meristem size regulation. To unveil if GH3.5 and GH3.6 regulate root meristem size by acting downstream of cytokinin we analyzed the root meristem size of these mutants upon cytokinin treatment. As previously reported, wild type plants treated with cytokinin, show a reduction of the meristem size [7,8], while *gh3.17–1* meristem is not affected [9]. Differently from *gh3.17–1*, *gh3.5–1* and *gh3.6–1* mutants showed a slight decrease in the number of meristematic cells when comparing the untreated and the cytokinin treated mutant plants (Figure 2C). These data indicate that the root meristem size of those mutants is only partially affected by cytokinin activity, suggesting that cytokinin regulates root meristem size also via GH3.5 and GH3.6. To further investigate the relation between GH3.17, GH3.5, and GH3.6 activities in controlling meristem size, we generated the *gh3.17–1;gh3.5–1* and *gh3.17–1;gh3.6–1* double mutants. Both the double mutants *gh3.17–1;gh3.5–1* and *gh3.17–1;gh3.6–1* showed a meristem size similar to that of the parental single mutants *gh3.17–1*, *gh3.5–1* and *gh3.6–1* (Figure 3), corroborating the idea that these GH3s act in the same pathway in regulating auxin levels to control meristem size. We, thus, inferred that cytokinin globally promotes the auxin inactivation process triggering *GH3.17*, *GH3.5*, and *GH3.6* expressions, and as a consequence, determining root meristem size.

## 3. Discussion

In plants, the hormone auxin is distributed as a gradient with morphogenetic properties, similarly to retinoic acid in animals [26,27,28,29]. Indeed, variations in auxin distribution profoundly change cell developmental programs [30]. In the root, an auxin maximum controls stem cell activities [30,31,32] while an auxin minimum establishes the position of the TZ, a cell boundary where stem cell daughters stop to divide and start to differentiate [9]. Indeed, differences in auxin contents between cells of the same tissue are translated into a developmental switch from proliferation to differentiation. The position of the auxin minimum in the root depends on the activity of the GH3.17 enzyme that specifically acts in the LRC tissue [9,17]. 

Here, we demonstrated that cytokinin supports TZ positioning and, hence, cell differentiation by controlling in the LRC the expression of multiple genes belonging to the *GH3* Group II gene family, such as *GH3.17*, *GH3.5*, and *GH3.6*. These GH3s conjugate auxin to different amino acids, thus, adjusting the levels of active auxin within each cell [9,18,19,21,22,23,24].

Cytokinin-dependent control of *GH3.17* expression [9] and the simultaneous activation of *GH3.5* and *GH3.6* gene expression (this work) highlights that auxin inactivation process strongly depends on cytokinin activity in the LRC. Interestingly, it has been already reported that a coordinated *GH3.5*, *GH3.6*, and *GH3.17* activity is necessary during hypocotyl elongation [33].

Although auxin negatively regulates its own levels by promoting *GH3.5* and *GH3.6* expression [25,34,35], *GH3.17* is not controlled by auxin itself [9]. Thus, *GH3.17* cytokinin-dependent control determines a change of auxin levels without suffering from any auxin feedback.

The data collected here show that the specific localized expression of three *GH3* Group II genes, regulating auxin inactivation in the LRC tissue, is crucial for meristem activity. Moreover, from these results, the LRC emerges as an important tissue where GH3-dependent auxin conjugation takes place and, hence, the site where the control of auxin levels is finely imposed in the root. Intriguingly, cytokinin-dependent *GH3.5* and *GH3.6* regulation happens in both LRC and vascular bundle. It will be interesting to know whether GH3.5 and GH3.6 are required in both of those tissues to control root meristem size. Further studies are required to address this crucial point. Nonetheless, the expression domain of the *GH3s* genes prompts the hypothesis that the control of auxin inactivation has to be confined to specific tissues rather than to the whole root to control root meristem size and, therefore, organ growth. 

## 4. Materials and Methods

### 4.1. Plant Material and Growth Conditions

The *Arabidopsis thaliana* ecotypes *Columbia-0* (*Col-0*) was used as a control because the *gh3.17–1* [9], *gh3.5–1* and *gh3.6–1* mutants are in *Col-0* background. *gh3.5–1* and *gh3.6–1* lines were obtained from the NASC collection (SALK_033434C and SALK_082530). Homozygous mutants from the Salk T-DNA were identified by PCR as described (http://signal.salk.edu/tdna primers.html). For growth conditions, *Arabidopsis* seeds were surface sterilized, and seedlings were grown on one-half strength Murashige and Skoog (MS) medium containing 0.8% agar at 22 °C in long-day conditions (16-h-light/8-h-dark cycle) as previously described [3].

*Arabidopsis* locus IDs from this article: *GH3.17* (AT1G28130), *GH3.5* (AT4G27260), *GH3.6* (AT5G54510) and *GH3.9* (AT2G47750).

### 4.2. Generation of GH3s Transgenic Plants

Standard molecular biology techniques and the Gateway system (Invitrogen) were used for the cloning procedures. For the *pGH3.5::GH3.5-GFP*, *pGH3.6::GH3.6-GFP*, and *pGH3.9::GH3.9-GFP* transgenic plants, the promoter sequences of *GH3.5* (2959 bp), *GH3.6* (1993 bp), *GH3.9* (2312 bp), and *GH3.17* (2128 bp) and genomic sequences of *GH3.5* (2189 bp), *GH3.6* (2244 bp), and *GH3.9* (2668 bp) were amplified from genomic DNA of *Arabidopsis thaliana Columbia* ecotype using specific primers (pGH3.5 FW 5’-TTTTTCATTGGATGTGAGGAA-3’, pGH3.5 REV 5’-GGTTTAAGAGAAAGAGAGAAGTCTGAG-3’, pGH3.6 FW 5’-AAAACCCATTAACAGCAGACG-3’, pGH3.6 REV 5’-CGTTTAGGTTTTGTGTTTAAAATTC-3’, pGH3.9 FW 5’-TGTCCTTGCAAGTGCAAAAT-3’, pGH3.9 REV 5’-TTCTCAGCTAACCCAAAGAAAG-3’, pGH3.17 FW 5’-GGGCGTTACGTATCAGGAAA-3’, pGH3.17 REV 5’-TGTCTGAAAGCAGACACAAACA-3’, gGH3.5 FW 5’-ATGCCTGAGGCACCAAAGAA-3’, gGH3.5 REV 5’-GTTACTCCCCCACTGTTTGTG-3’, gGH3.6 FW 5’-ATGCCTGAGGCACCAAAG-3’, gGH3.6 REV 5’-GTTACTCCCCCATTGCTTGT-3’, gGH3.9 FW 5’-ATGGATGTAATGAAGCTTGATCA-3’, gGH3.9 REV 5’-TGGAACCCAAGTCGGGTC-3’) and cloned in a *pDONOR-P4P1* and *pDONOR-221* vectors: *pDONOR-P4P1-pGH3.5*, *pDONOR-P4P1-pGH3.6*, *pDONOR-P4P1-pGH3.9*, and *pDONOR-P4P1-pGH3.17*, promoter sequences, respectively, *pDONOR-221-pGH3.5*, *pDONOR-221-pGH3.6*, and *pDONOR-221-pGH3.9*, genomic sequences, respectively. The LR reactions were then conducted by using the *pDONOR-P4P1-pGH3.5*/*pGH3.6*/*pGH3.9*, the *pDONOR221-gGH3.5*/*gGH3.6*/*gGH3.9* and a *pDONORP2P3-GFP* vectors.

For *pGH3.5–3xYFP*, *pGH3.6–3xYFP*, *pGH3.9–3xYFP*, and *pGH3.17–3xYFP* transgenic plants, the promoter sequences of *GH3.5*, *GH3.6*, *GH3.9*, and *GH3.17* cloned in the *pDONOR-P4P1* vectors, as described above, were used. The LR reactions were then conducted by using the *pDONOR-P4P1- pGH3.5*/*pGH3.6*/*pGH3.9*/*pGH3.17*, a *pDONOR221–3xYFP*, and *pDONORP2P3-NOST2* vectors [36]. The obtained LR products were then sub-cloned in the Gateway *pBm43GW* destination vector. Plasmids were transformed into *Col-0* plants by floral dipping [37]. Each expression domain of the T2 generation of the *3xYFP* transcriptional fusion lines was analyzed to verify homogeneous expression. Each transgenic line revealed the same YFP expression pattern.

### 4.3. Hormonal Treatments

Five days after germination (dag) seedlings were transferred onto solid one-half MS medium containing 0.025% DMSO solvent (mock condition) or onto solid medium containing a final concentration of 5 μM trans-Zeatin (tZ, Duchefa) dissolved in DMSO (0.025% final concentration). A twenty-two-hour hormone treatment was used for meristem size analysis in response to cytokinin and a four-hour hormone treatment was used for *GH3s* transcriptional reporter lines expression analysis. 

### 4.4. Bright Field and Confocal Microscopy Analysis

Differential interference contrast (DIC) with Nomarski technology microscopy (Zeiss Axio Imager A2 microscope) was used to count meristem cell number with bright field microscopy. Root meristem size of each plant was measured based on the number of cortex cells in a file extending from the quiescent center to the first elongated cortex cell excluded [3]. Plants were mounted in a chloral hydrate solution [3]. Confocal images were obtained using a Zeiss LSM 780 confocal laser scanning microscope. For confocal laser scanning analysis, a propidium iodide 10 μM staining was used. For each experiment, two biological replicates were performed, and the number of samples analyzed were reported in the relative figure legend. Results were comparable in all experiments. The statistical significance was determined by Student’s t-test (http://graphpad.com/quickcalcs/ttest2.cfm), data were reported in the relative figure legend.

### 4.5. GH3s Reporter Lines Fluorescence Quantification

The fluorescence intensity of *pGH3.5–3xYFP*, *pGH3.6–3xYFP* and *pGH3.17–3xYFP* lines untreated and treated with cytokinin 5 μM for four hours (Figure 1) was quantified as reported in [3]. Mean Grey Value of YFP channel of confocal laser scanning microscope images was measured with the software *ImageJ* (https://imagej.nih.gov/ij/). Fluorescence signal was measured taking into consideration the same area for untreated and treated lines (length 550 µm × width 187 µm) starting from the tip of the root. Student’s t-test was used to determine the statistical significance (http://graphpad.com/quickcalcs/ttest2.cfm) as reported in the relative figure legend.

### 4.6. Statistical Analysis Criteria

All the experiments were performed with a number of samples large enough to ensure the statistical significance of the analysis, as reported in corresponding figure legends. Representative sample pictures of the experiments were chosen in all figures.
